# Molecular mechanism of G_1_ arrest and cellular senescence induced by LEE011, a novel CDK4/CDK6 inhibitor, in leukemia cells

**DOI:** 10.1186/s12935-017-0405-y

**Published:** 2017-03-06

**Authors:** Yan-Fang Tao, Na-Na Wang, Li-Xiao Xu, Zhi-Heng Li, Xiao-Lu Li, Yun-Yun Xu, Fang Fang, Mei Li, Guang-Hui Qian, Yan-Hong Li, Yi-Ping Li, Yi Wu, Jun-Li Ren, Wei-Wei Du, Jun Lu, Xing Feng, Jian Wang, Wei-Qi He, Shao-Yan Hu, Jian Pan

**Affiliations:** 1 Institute of Pediatrics, Children’s Hospital of Soochow University, Suzhou, China; 2 Department of Hematology and Oncology, Children’s Hospital of Soochow University, Suzhou, China; 3 CAM-SU Genomic Resource Center, Soochow University, Suzhou, China

**Keywords:** LEE011, Leukemia, CDK4/6, Cellular senescence, Arraystar Human LncRNA array

## Abstract

**Background:**

Overexpression of cyclin D1 dependent kinases 4 and 6 (CDK4/6) is a common feature of many human cancers including leukemia. LEE011 is a novel inhibitor of both CDK4 and 6. To date, the molecular function of LEE011 in leukemia remains unclear.

**Methods:**

Leukemia cell growth and apoptosis following LEE011 treatment was assessed through CCK-8 and annexin V/propidium iodide staining assays. Cell senescence was assessed by β-galactosidase staining and p16^INK4a^ expression analysis. Gene expression profiles of LEE011 treated HL-60 cells were investigated using an Arraystar Human LncRNA array. Gene ontology and KEGG pathway analysis were then used to analyze the differentially expressed genes from the cluster analysis.

**Results:**

Our studies demonstrated that LEE011 inhibited proliferation of leukemia cells and could induce apoptosis. Hoechst 33,342 staining analysis showed DNA fragmentation and distortion of nuclear structures following LEE011 treatment. Cell cycle analysis showed LEE011 significantly induced cell cycle G_1_ arrest in seven of eight acute leukemia cells lines, the exception being THP-1 cells. β-Galactosidase staining analysis and p16^INK4a^ expression analysis showed that LEE011 treatment can induce cell senescence of leukemia cells. LncRNA microarray analysis showed 2083 differentially expressed mRNAs and 3224 differentially expressed lncRNAs in LEE011-treated HL-60 cells compared with controls. Molecular function analysis showed that LEE011 induced senescence in leukemia cells partially through downregulation of the transcriptional expression of MYBL2.

**Conclusions:**

We demonstrate for the first time that LEE011 treatment results in inhibition of cell proliferation and induction of G_1_ arrest and cellular senescence in leukemia cells. LncRNA microarray analysis showed differentially expressed mRNAs and lncRNAs in LEE011-treated HL-60 cells and we demonstrated that LEE011 induces cellular senescence partially through downregulation of the expression of MYBL2. These results may open new lines of investigation regarding the molecular mechanism of LEE011 induced cellular senescence.

**Electronic supplementary material:**

The online version of this article (doi:10.1186/s12935-017-0405-y) contains supplementary material, which is available to authorized users.

## Background

Acute leukemia is the most common pediatric malignancy constituting more than 30% of all childhood cancers [1]. Approximately 300 important genes have been reported to be altered in hematologic malignancies. Pediatric acute myeloid leukemia (AML) accounts for more than 50% of pediatric acute leukemia patient deaths. More effective therapeutic strategies are needed to improve prognosis. Recently, the potential therapeutic application of CDK4/6 inhibitors in a range of cancers has been considered.

The proteins encoded by CDK4 and 6 are members of the Ser/Thr protein kinase family [2]. Both CDK4 and 6 are important for cell cycle regulation, specifically G_1_ phase progression, with their activity strictly restricted to the G_1_-S phase [3–5]. Mutations in these genes have been found to be significantly associated with tumorigenesis of several cancers [6, 7]. It is now believed that the vast majority of human tumors exhibit deregulation of the CDK4/6-cyclin D-INK4-RB pathway through multiple mechanisms [8–10]. CDK4/6 amplification or overexpression has also been observed in a range of tumors, including lymphomas, melanomas, gliomas, sarcomas, carcinomas of the breast and leukemias. For example, CDK6 promoter related chromosomal translocation leads to CDK6 overexpression, which has been reported in B cell lymphocytic leukemias and splenic marginal zone lymphoma [11, 12].

Several pharmacological inhibitors of CDK4/6 have been developed and many are currently being tested in clinical trials. One CDK4/6 selective inhibitor, PD-0332991, causes G_1_ arrest and growth inhibition in xenograft models of human tumor cell lines including breast, ovary, lung and multiple myeloma. Another CDK4/6 inhibitor, LY2835219, has been reported to inhibit CDK4 and 6 at very low concentrations, resulting in proliferation inhibition and G_1_ cell cycle arrest [13]. GCS-100 is a non-selective CDK6 inhibitor which induces inhibition of proliferation and apoptosis in myeloma cell lines [14]. KBH-A42 is a new synthetic histone deacetylase inhibitor which can effectively inhibit the growth of several cancer cells [15]. Results suggest that the molecular mechanism of KBH-A42 mediated cell cycle arrest may be the result of the down regulation of CDK4 and CDK6 [16].

LEE011 is a recently developed CDK4/6 inhibitor [17]. LEE011 has shown antiproliferative effects in a panel of human cancer cell lines and primary tumor xenografts. For example, oral administration of LEE011 to mice bearing human liposarcoma xenografts resulted in approximately 50% reduction in tumors [18]. Further studies have shown that treatment with LEE011 significantly reduced cell proliferation in 12 of 17 human neuroblastoma cell lines [17, 19]. To date, the molecular function of LEE011 in leukemia is unclear. In this study the antitumor effect of LEE011 was evaluated in leukemia cells to further characterize its preclinical efficacy and molecular mechanism.

## Methods

### Cell and culture conditions

Leukemia cell lines HL-60, MV4-11, U937 and K562 were obtained from the American Type Culture Collection (ATCC). CCRF, 697 and SHI-1 cell lines (gifts from The Cyrus Tang Hematology center of Soochow University). NB4 and THP-1 cell lines (gifts from Hematology Institute of Soochow University). All cell lines were maintained at 37 °C in the RPMI 1640 (GibcoR, Life Technologies, Carlsbad, CA, USA) supplemented with 10% fetal bovine serum (Invitrogen, Life Technologies, Carlsbad, CA, USA). LEE011 (Cat: S7440 Selleck Chemicals, West Paterson, NJ, USA) was dissolved in DMSO (Cat: D4540 Sigma-Aldrich, St. Louis, MO, USA).

### Patients and samples

Bone marrow specimens were obtained at the time of diagnosis from 5 pediatric AML and 5 ALL patients between 2014 and 2015. Ethical approval was provided by the Children’s Hospital of Soochow University Ethics Committee (No. SUEC2013-022), and written informed consent was obtained from the parents or guardians. They will be given the opportunity to withdraw from the research at any time prior to the publication of the research findings. The matter of how data will be collected and stored, with reference to the data protection legislation will be clarified for participants, with information being stored in locked cabinets or on IT hardware protected with the highest security software. The main clinical and laboratory features of the patient cohort are summarized in Tables 1 and 2. Bone marrow mononuclear cells (BMNCs) were isolated using Ficoll solution within 2 h after bone marrow samples harvested.

**Table 1:** Pathologic features and inhibition of cell growth by LEE011 in primary culture cells of pediatric ALL

	Gender	Age	Diagnosis	ALL typing	Chromosome analysis	Fusion gene	CDK6	IC50 μM
1	F	5	ALL	B	46, XX	Not detected	Positive	2.14
2	M	4	ALL,	B	46, XY	TEL/AML1(+)	Positive	1.73
3	M	3	ALL	B	ALL/53–54, XY, +4, +6, +10, 12p+, +14, +17, +18, +20, +21	Not detected	Positive	14.68
4	F	4	ALL	B	46, XX	Not detected	Positive	2.68
5	F	4	ALL	B	ALL/53–55XX, +X, 1q+, +4, +6, +10, +11, +15, +17, +21	Not detected	Positive	11.52

**Table 2:** Pathologic features and inhibition of cell growth by LEE011 in primary culture cells of pediatric AML

	Gender	Age	Diagnosis	AML typing	Chromosome analysis	Fusion gene	CDK6	IC50 μM
1	F	9	AML	M4	46, XX	FLT3-ITD	Positive	2.54
2	F	3	AML	M4	46, XX, inv(16)(p13q22)	CBF/MYH11	Positive	8.46
3	M	4	AML	M5b	46, XY, −2, +10, t(10;10)(p13;q23)	MLL/AF10	Positive	1.94
4	M	12	AML	M2a	45, X, −Y, t(8;21)(q22;q22)	AML/ETO	Positive	5.04
5	F	1	AML	M4	46, XX, inv(16)(p13q22)	46, XY, inv(16)(p13q22)	Positive	2.98

### Cell proliferation

Cell proliferation analysis was almost same as introduced before [20]. Leukemia cells were incubated with DMSO, or increasing concentrations of LEE011 (0.05–80 μM) for 24 h. CCK8 Kit (Dojindo Molecular Technologies, Japan) was used to analyze the cell survival rate. The IC50 of LEE011 inhibitor was calculated by Graph Prism software.

### Cell cycle analysis

Cell cycle analysis was also introduced before [20]. Leukemia cells were collected, fixed, incubated with 1.5 μmol/l propidium iodide (P4170, Sigma-Aldrich, St. Louis, MO, USA) and 25 μg/ml RNase A The samples (1 × 10^4^ cells) and were analyzed with a Beckman Gallios™ Flow Cytometer. Then these data was analyzed with cell cycle software (MultiCycle for Windows).

### Apoptosis assay

Apoptosis assay was according to the manual operation of BD Annexin V Staining Kit (Cat: 556420, BD Biosciences, Franklin Lakes, NJ, USA). All the details have been introduced before [20, 21].

### Hoechst 33,342 staining analysis

Cells were seeded into 6-well plates, and then treated with LEE011 (2 or 5 μM) and cultured at 37 °C for 24 h, stained with 0.1 µg/ml Hoechst 33,342 (Sigma, St. Louis, MO, USA) for 5 min, then observed with filters for blue fluorescence under fluorescence microscopy (OLYMPUS IX71; Olympus Corporation, Tokyo, Japan). Abnormal nuclear cells were counted between the RO3280 treatment group and DMSO control group [22].

### Cell senescence β-galactosidase staining analysis

Leukemia cells were seeded into 6-well plates, and then treated with LEE011 (2 or 5 μM) and cultured at 37 °C for 24–72 h, senescence β-galactosidase staining analysis was according to the manufacture of senescence β-galactosidase staining kit (Beyotime Corporation, C0602, Jiangsu, China). Staining cells were photographed with microscopy (OLYMPUS IX71; Olympus Corporation, Tokyo, Japan). Positive staining cells were counted between the LEE011 treatment group and DMSO control group.

### Analyze the genes and LncRNAs expression profiles related with LEE011

HL-60 cells were treated with 1 μM LEE011 and control group cells were treated with the same volume of DMSO 24 h later. Human LncRNA array analysis was performed by KangChen Bio-tech, Shanghai P. R. China. And experimental details were introduced by Yu et al. [23]. RNA purification and analysis was introduced as before [22].

### Gene ontology analysis and KEGG pathway analysis the genes expression profiles related with LEE011

Gene ontology (GO) analysis introduced before [24] is a functional analysis that associates differentially expressed mRNAs with GO categories (http://www.geneontology.org). The lower the *P* value is, the more significant the GO term (a *P* ≤ 0.05 is recommended). Pathway analysis is a functional analysis that maps genes to Kyoto encyclopedia of genes and genomes (KEGG) pathways (http://www.genome.jp/kegg/) was introduced before [25]. The *P* value (EASE-score, Fisher *P* value or Hypergeometric *P* value) denotes the significance of the pathway correlated to the conditions. The lower the *P* value is, the more significant the correlation (the recommend *P* value cut-off is 0.05).

### Western blot analysis

For western blot analysis, protocol is introduced before [26]. Blots were blocked and then probed with antibodies against Caspase 3 (Cat: 9661S 1:1000, Cell Signaling Technology, Inc. Danvers, MA, USA), Caspase 9 (Cat: 4501S 1:1000, Cell Signaling Technology, Inc. Danvers, MA, USA), PARP (Cat: 9542S, 1:1000, Cell Signaling Technology, Inc. Danvers, MA, USA), CDK6 (Cat: 13331S 1:1000, Cell Signaling Technology, Inc. Danvers, MA, USA), CDK4 (Cat: 12790S 1:1000, Cell Signaling Technology, Inc. Danvers, MA, USA), Cyclin D1 (Cat: 2978S 1:1000, Cell Signaling Technology, Inc. Danvers, MA, USA), Cyclin D2 (Cat: 3741S 1:1000, Cell Signaling Technology, Inc. Danvers, MA, USA), RB (Cat: 9313S 1:1000, Cell Signaling Technology, Inc. Danvers, MA, USA), p-RB (Cat: 8516S 1:1000, Cell Signaling Technology, Inc. Danvers, MA, USA), KIF20A (Cat: ab85644 1:1000, Abcam Trading (Shanghai) Company Ltd. Pudong, Shanghai, China), PLK1 (Cat: 4535S 1:1000, Cell Signaling Technology, Inc. Danvers, MA, USA), MYBL2 (Cat:BA3860 1:1000, BOSTER (Wuhan) Company Ltd. Wuhan, Chin), p16^INK4a^ (Cat: ab189302 1:1000, Abcam Trading (Shanghai) Company Ltd. Pudong, Shanghai, China), p21 ^Waf1/Cip1^ (Cat: 2946S 1:1000, Cell Signaling Technology, Inc. Danvers, MA, USA),GAPDH (1:5000, Sigma, St. Louis, MO, USA).

### Real-time PCR analysis certification of dyes-regulated genes in LEE011-treated HL-60 cells

Quantitative real-time PCR was performed to determine the expression levels of dyes-regulated genes in 1 μM LEE011-treated HL-60 cells. Real-time PCR analysis was introduced before [26]. cDNA synthesis was performed on 4 μg of RNA in a 10 μl sample volume using SuperScript II reverse transcriptase (Invitrogen Co., NY, USA) as recommended by the manufacturer. Reactions were run on Light cycler 480 using the universal thermal cycling parameters. The real time PCR primers used to quantify GAPDH expression were: F: 5′-AGAAGGCTGGGGCTCATTTG-3′ and R: 5′-AGGGGCCATCCACAGTCTTC-3′;

CR1L were F: 5′-GTCCTCCTTCTCCGATCAATGC-3′ and R: 5′-CTTAGCACTTGTCCAGACTGAG-3′; TCP11L2 were F: 5′-CTAAATGCTGACCCTCCTGAGT-3′ and R: 5′- GCCACCGGGAGTGAGAAAA-3′; CR1 were F: 5′-AGAGGGACGAGCTTCGACC-3′ and R: 5′-TCAGGACGGCATTCGTACTTT-3′; AMICA1 were F: 5′-GTTTCCCCGCCTGAGCTAAC-3′ and R: 5′-TTCTGGAAGCGCCCAATAGG-3′; MCM10 were F: 5′-AAGCCTTCTCTGGTCTGCG-3′ and R: 5′-CTGTGGCGTAACCTTCTTCAA-3′; CDK1 were F: 5′-AAACTACAGGTCAAGTGGTAGCC-3′ and R: 5′-TCCTGCATAAGCACATCCTGA-3′; DLGAP5 were F: 5′-AAGTGGGTCGTTATAGACCTGA-3′ and R: 5′-TGCTCGAACATCACTCTCGTTAT-3′; KIF20A were F: 5′-TGCTGTCCGATGACGATGTC-3′ and R: 5′-AGGTTCTTGCGTACCACAGAC-3′; S100A8 were F: 5′-CATGCCGTCTACAGGGATGA-3′ and R: 5′- GACGTCTGCACCCTTTTTCC-3′; IL8 were F: 5′-GAATGGGTTTGCTAGAATGTGATA-3′ and R: 5′-CAGACTAGGGTTGCCAGATTTAAC-3′; PLK1 were F: 5′- CTCAACACGCCTCATCCTC-3′ and R: 5′-GTGCTCGCTCATGTAATTGC-3′; MYBL2 were F: 5′-TGCCAGGGAGGACAGACAAT-3′ and R: 5′-CTGTACCGATGGGCTCCTGTT-3′; PADI4 were F: 5′-AGTGGCTTGCTTTCTTCTCCTGTG-3′ and R: 5′-AGCAGAACTGAGTGTGCAGTGCTA-3′. Expression of genes was normalized to endogenous GAPDH expression.

Cluster analysis of the data was performed with gene cluster from the real-time PCR arrays. For gene expression quantification, we used the comparative Ct method. First, gene expression levels for each sample were normalized to the expression level of the housekeeping gene encoding glyceraldehyde 3-phosphate dehydrogenase (GAPDH) within a given sample (−ΔCt). The relative expression of each gene was calculated using the equation: 10^6^*Log_2_ (−ΔCt). Gene expression between the DMSO and the LEE011 samples were analyzed using Multi Experiment View (MEV) cluster software.

### Interfering expression of LEE011 target genes in leukemia cells with RNAi lentivirus

RNAi lentivirus was purchased from Shanghai Genechem Co., Ltd. (http://www.genechem.com.cn). RNAi products target-specific lentivirus designed to knockdown MYBL2 expression; sequences are 1# 5-CAGATCAGAAGTACTCCAT-3; for KIF20A, sequences are 1# 5- CAGAAGAATATAAGGCTGT-3; for PLK1, sequences are 1# 5-CAACCAAAGUCGAAUAUGA-3. The control sequence is 5-TTCTCCGAACGTGTCACGT-3. Lentivirus infection was according to the manufacture of Shanghai Genechem Co., Ltd. at a final concentration of 100–200 MOI (multiplicity of infection). Interference efficiency was measured by western blot at 3 days after transfection. The rest cells were harvested for further analysis.

### Statistical analysis

Each experimental condition was performed for three times, and these replicates were presented in results and figures. All values are presented as mean ± SEM. Student’s paired t test was applied to reveal statistical significances. P values less than 0.05 were considered significant. Statistical analyses were performed using SPSS Software for Windows (version 11.5; SPSS, Inc., Chicago, IL, USA).

## Results

### Inhibitory effect of LEE011 on acute leukemia cell growth

**Fig. 1:**
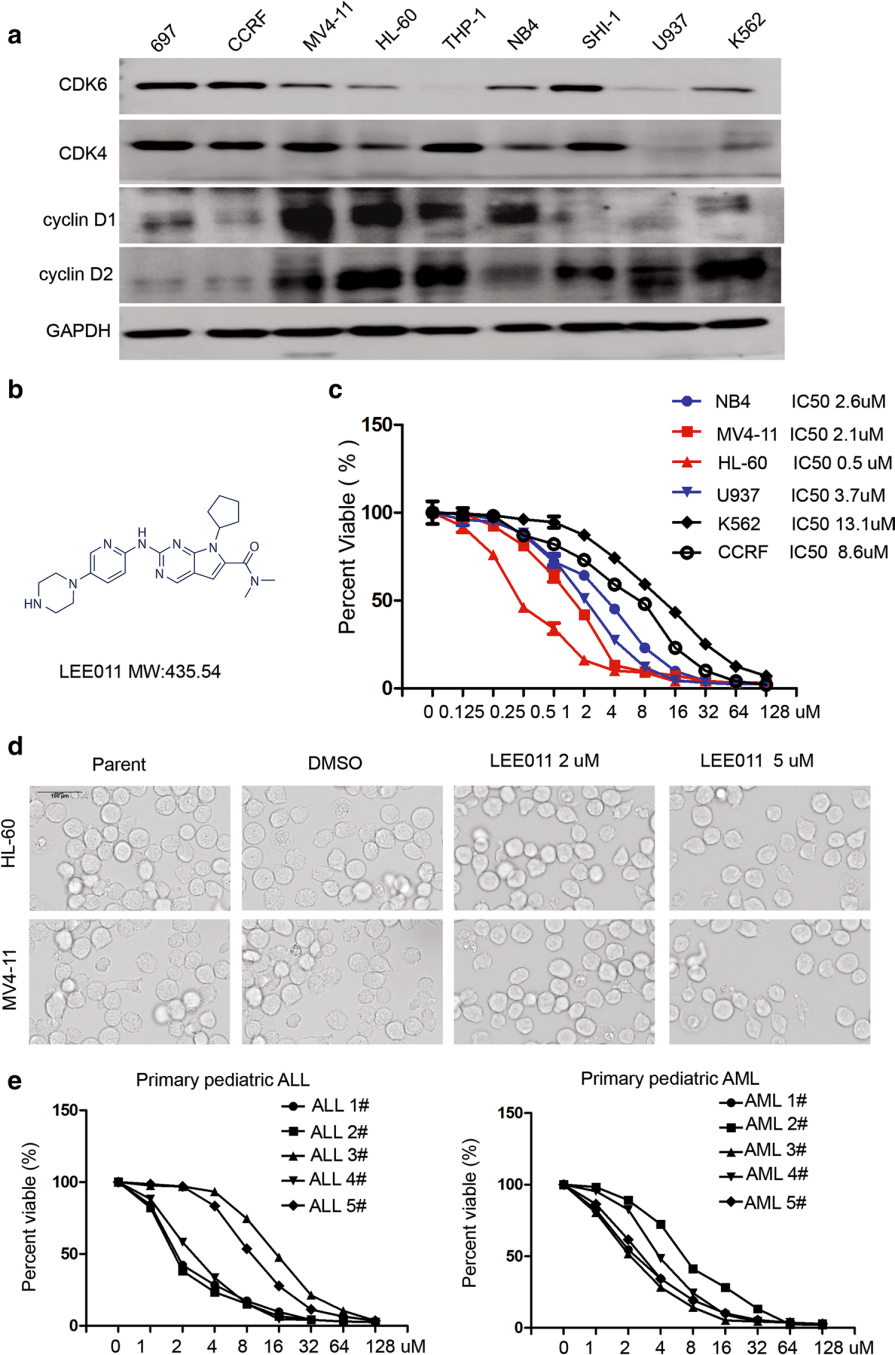
Inhibitory effect of LEE011 on leukemia cell growth. **a** Western blot analysis showing the expression of CDK6, CDK4, Cyclin D1 and Cyclin D2 in nine leukemia cell lines. Our results indicated that there is positive correlation between IC50 and the expression of Cyclin D1. **b** Molecular structure of LEE011. **c** Proliferation and IC50 analysis of LEE011 in six leukemia cells. IC50s: U937 3.7 µM, HL60 0.5 µM, NB4 2.6 µM, MV4-11 2.1 µM, K562 13.1 µM and CCRF 8.6 µM. **d** Micrographs of HL-60 and MV4-11 cells treated with LEE011 (2 and 5 µM) or DMSO. **e** The IC50 of LEE011 in primary ALL and AML cells was also analyzed. In primary ALL cells, the IC50 of LEE011 was 1.73–14.68 µM; in primary AML cells the IC50 of LEE011 was 1.94–8.46 µM. All experiments were performed in quadruplicate. *P < 0.05, **P < 0.01

Western blot analysis showed that expression of CDK6 was very high in seven of nine of the leukemia cell lines investigated, the exceptions being THP-1 and U937 cells (Fig. 1a). CDK4, Cyclin D1 and Cyclin D2 were also examined with Western blot analysis. Our results indicated that there is positive correlation between IC50 and the expression of Cyclin D1. LEE011 is novel CDK4/CDK6 inhibitor with very high specificity (Fig. 1b). LEE011 treatment resulted in inhibition of proliferation of leukemia cells in a dose-dependent manner (Fig. 1c). Cell morphology and cell number of HL-60 and MV4-11 cells were analyzed; there were changes in cell number following LEE011 treatment at concentrations of 2 and 5 µM but no alteration in cellular morphology (Fig. 1d). To better understand the efficacy of LEE011, IC50 values of LEE011 in primary acute lymphoblastic leukemia (ALL) and AML cells were analyzed (Tables 1, 2). Our results showed that in primary ALL cells, the IC50 of LEE011 was 1.73–14.68 µM and in primary AML cells the IC50 of LEE011 was 1.94–8.46 µM (Fig. 1e). These results confirm that LEE011 is an effective anti-leukemia inhibitor.

### LEE011 can induce apoptosis in leukemia cells

**Fig. 2:**
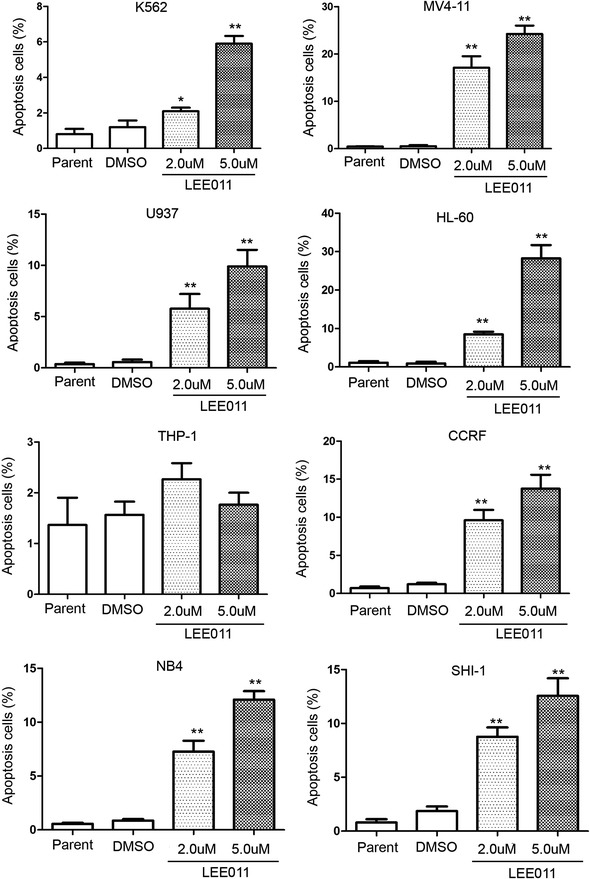
Analysis of apoptosis in leukemia cells induced by LEE011. Annexin V staining of cells following 48-h treatment with LEE011 at 2 or 5 µM compared with DMSO controls. Following 5-µM LEE011 treatment, the K562 apoptotic cell percentage was 5.9 ± 0.75 vs. 1.2 ± 0.66% for the DMSO group, P = 0.001; in MV4-11 cells, the apoptotic cell percentage was 24.2 ± 3.06 vs. 0.53 ± 0.40% for the DMSO group, P = 0.005; in U937 cells, the apoptotic cell percentage was 9.9 ± 2.81 vs. 0.57 ± 0.42% for the DMSO group, P = 0.027; in HL-60 cells, the apoptotic cell percentage was 28.23 ± 6.01 vs. 0.9 ± 0.8% for the DMSO group, P = 0.015; in THP-1 cells, the apoptotic cell percentage was 1.76 ± 0.4 vs. 1.56 ± 0.45% for the DMSO group, P = 0.59; in CCRF cells, the apoptotic cell percentage was 13.77 ± 3.16 vs. 1.2 ± 0.36% for the DMSO group, P = 0.019; in NB4 cells, the apoptotic cell percentage was 12.1 ± 1.35 vs. 0.86 ± 0.25% for the DMSO group, P = 0.004; and in SHI-1 cells the apoptotic cell percentage was 12.6 ± 2.81 vs. 1.87 ± 0.75% for the DMSO group, P = 0.017. These analyses were repeated three times. *P < 0.05; **P < 0.01

**Fig. 3:**
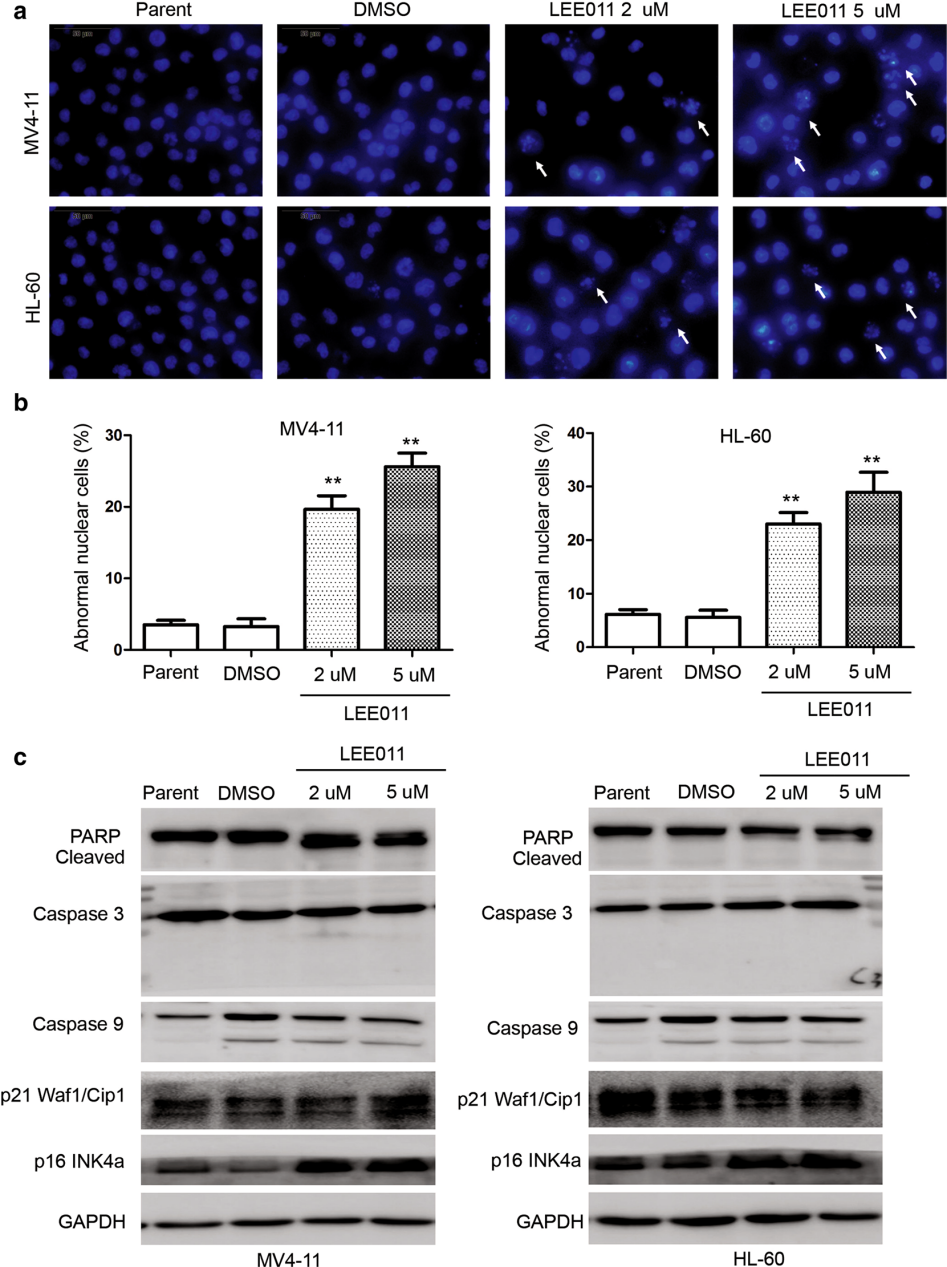
Analysis of apoptosis and cellular senescence markers induced by LEE011 in leukemia cells. **a** Hoechst 33,342 staining analysis showing cells treated with LEE011 at 2 and 5 µM, demonstrates an increase in cells with DNA fragmentation and abnormal nuclear structure following 48-h LEE011 treatment. **b** The number of cells with abnormal nuclear structure was calculated in each group. For MV4-11 cells, the proportion with abnormal nuclear structure in the 5-µM treatment group was 28.93 ± 6.50% vs. DMSO group 5.60 ± 2.29%, P = 0.0016; for HL-60 cells, the respective values were 25.60 ± 3.30 vs. 3.27 ± 1.84%, P = 0.0013. **P < 0.01. **c** Western blotting of molecular markers of apoptosis, including PARP, caspase-3 and caspase-9, and molecular markers of cellular senescence, p16^INK4a^ and p21^Waf1/Cip1^. Upregulation of p16^INK4a^ was observed in the LEE011-treatment groups for both MV4-11 and HL-60 cells

We investigated apoptosis in leukemia cells following LEE011 treatment. Cells treated with LEE011 at 2 and 5 µM for 48 h showed more apoptotic features when compared to controls in seven leukemia cell lines, the exception being THP-1 (Fig. 2). To further demonstrate whether LEE011 causes apoptosis in leukemia cells, we assessed the expression and cleavage of the apoptosis markers PARP, caspase-3 and caspase-9 by western blot. After 48-h treatment with 2 and 5 µM LEE011, an increase in cleaved PARP was observed in the LEE011 treatment group for both MV4-11 and HL-60 cells (Fig. 3c).

Hoechst 33,342 staining analysis showed that DNA fragmentation and an increase in cells with nuclear abnormalities were observed after 24-h LEE011 treatment (Fig. 3a). Abnormal nuclear structure in cells increased significantly compared with DMSO treated control cells in both HL-60 and MV4-11 cell lines (Fig. 3b). The proportion of MV4-11 cells with abnormal nuclear structure in the 5-µM treatment group was 28.93 ± 6.50 vs. 5.60 ± 2.29% for the DMSO group (P = 0.0016); in HL-60 cells, 25.60 ± 3.30% of cells had abnormal nuclear structure in the 5-µM treatment group, compared with 3.27 ± 1.84% in the DMSO group (P = 0.0013).

### LEE011 induced G_1_ arrest and cellular senescence in leukemia cells

**Fig. 4:**
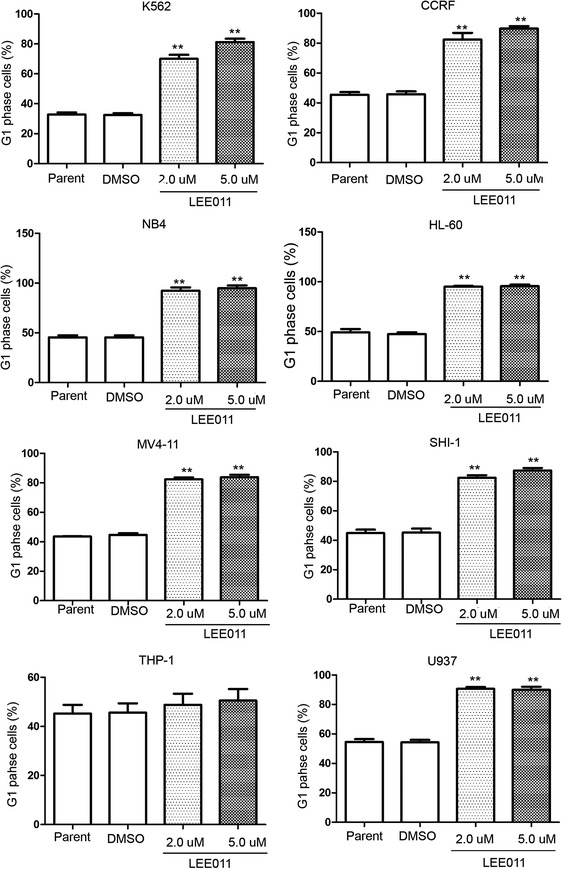
Cell cycle analysis of leukemia cells treated with LEE011. Cell cycle analysis showing cells treated with LEE011 at 2 or 5 µM for 48 h, in eight leukemia cell lines. LEE011 significantly induced cell cycle G_1_ arrest. The G_1_ phase cell percentage following 5-µM LEE011 treatment of K562 cells was 81.23 ± 3.84% vs. DMSO group 32.46 ± 2.21%, P < 0.01. The G1 phase cell percentage in CCRF cells treated with 5 µM LEE011 was 89.83 ± 2.67% vs. DMSO group 45.80 ± 3.24%, P < 0.01. The G_1_ phase cell percentage in NB4 cells treated with 5 µM LEE011 was 94.79 ± 4.93% vs. DMSO group 45.59 ± 3.12%, P < 0.01. The G_1_ phase cell percentage in HL-60 cells treated with 5 µM LEE011 was 95.50 ± 2.97% vs. DMSO group 47.40 ± 3.00%, P < 0.01. The G_1_ phase cell percentage in MV4-11 cells treated with 5 µM LEE011 was 83.82 ± 2.81% vs. DMSO group 44.66 ± 1.90%, P < 0.01. The G_1_ phase cell percentage in SHI-1 cells treated with 5 µM LEE011 was 87.39 ± 2.80% vs. DMSO group 45.25 ± 4.61%, P < 0.01. The G_1_ phase cell percentage in THP-1 cells treated with 5 µM LEE011 was 50.51 ± 8.17% vs. DMSO group 45.64 ± 6.46%, P = 0.466. The G_1_ phase cell percentage in U937 cells treated with 5 µM LEE011 was 89.99 ± 3.54% vs. DMSO group 54.26 ± 2.92%, P < 0.01. **P < 0.01

**Fig. 5:**
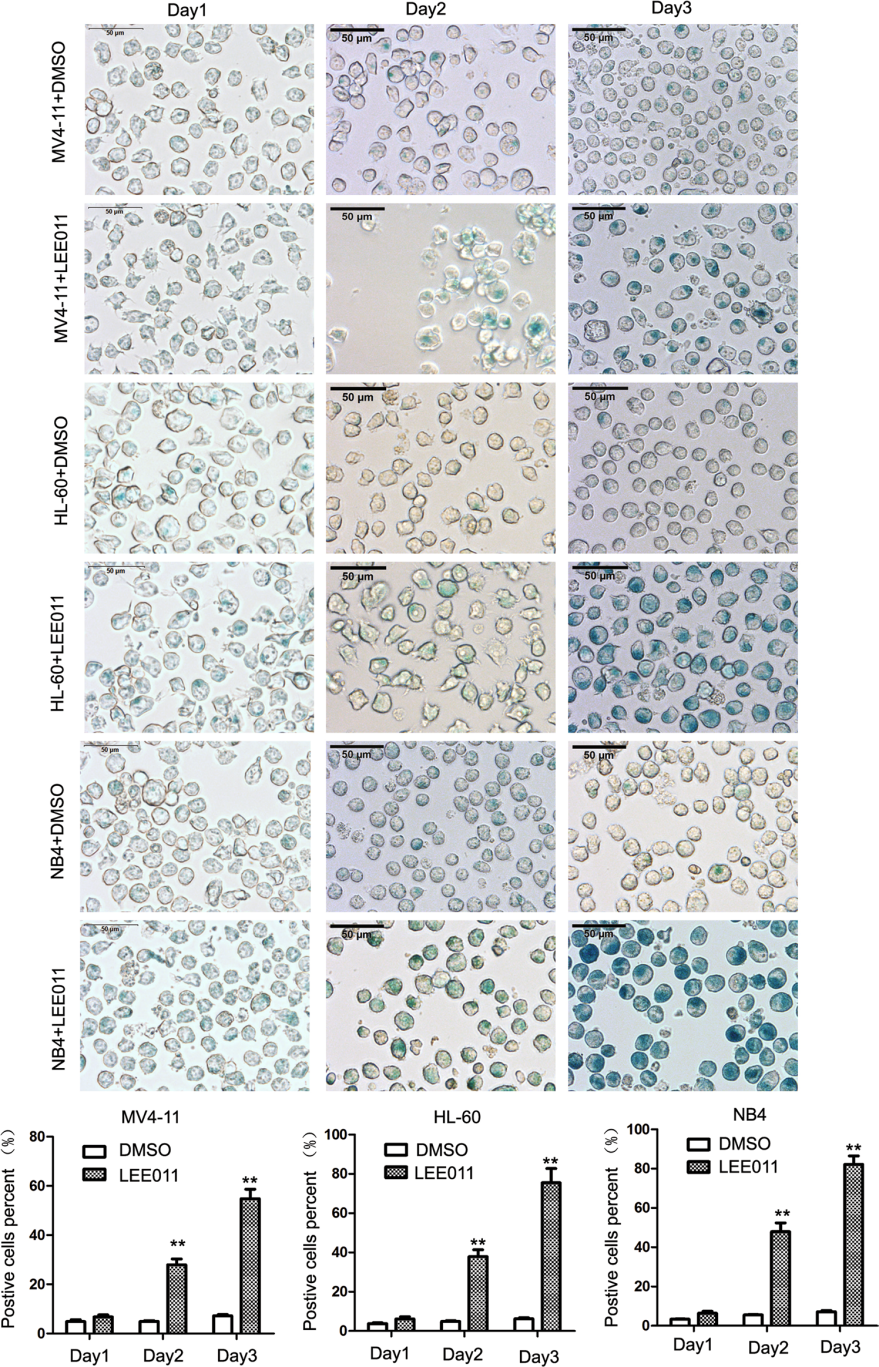
β-Galactosidase staining analysis of leukemia cells treated with LEE011. Leukemia cells were treated with 2.0 µM LEE011 for 24–72 h before analysis; more cells stained β-galactosidase positive in the LEE011-treatment group compared with the DMSO control group. For MV4-11 cells, 3-days-treated LEE011 group positive cells were 54.77 ± 6.68% vs. DMSO group 7.17 ± 0.95%, P = 0.006; in HL60 cells, 3-days-treated LEE011 group positive cells were 75.43 ± 12.67% vs. DMSO group 6.20 ± 1.00%, P = 0.0011; in NB4 cells, 3-days-treated LEE011 group positive cells were 82.10 ± 7.55% vs. DMSO group 7.17 ± 1.12%, P = 0.003. **P < 0.01

Cell cycle analysis was undertaken on cells treated with LEE011 at 2 and 5 µM for 48 h (Additional files 1, 2). LEE011 significantly induced cell cycle G_1_ arrest in acute leukemia cells except THP-1 cells (Fig. 4). Cell senescence β-galactosidase staining analysis was used in three leukemia cell lines: MV4-11, HL-60 and NB4. These cells were treated with LEE011 at 2 µM for 24–72 h before analysis (Fig. 5). More cells were β-galactosidase staining-positive following LEE011 treatment compared to DMSO controls. In MV4-11 and HL-60 cells, cell senescence marker p16^INK4a^ was upregulated significantly when the cells were treated with LEE011 for 48 h (Fig. 3c).

### Microarray analysis of genes and LncRNA expression profiles in LEE011-treated HL-60 cells

**Fig. 6:**
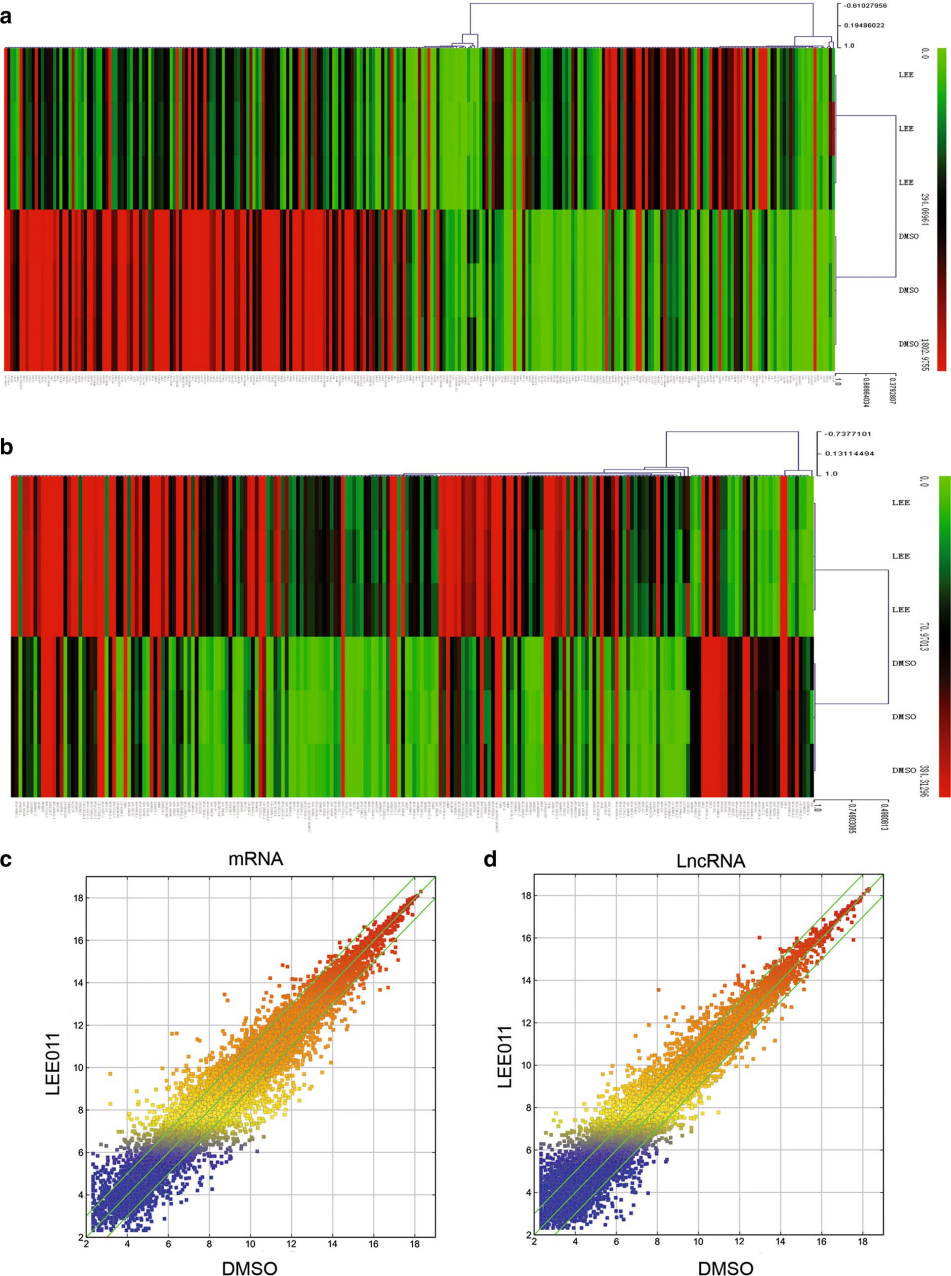
Microarray analysis of gene and LncRNA expression profiles in LEE011-treated HL-60 cells. The Arraystar Human LncRNA 8 × 60 k v3.0 1 microarray was used to identify mRNA and lncRNA expression profiles in LEE011-treated HL-60 cells compared with controls. **a** Hierarchical clustering analysis of the 116 and 155 significantly up- and downregulated mRNAs (≥fivefold) in LEE011-treated HL-60 cells. **b** Hierarchical clustering analysis of the differently expressed lncRNAs with a fold-change ≥5 in LEE011-treated HL-60 cells. **c** *Scatter-plot* showing mRNA expression variation between the control group and LEE011 treated HL-60 cells. The *green lines* are fold-change lines (the default fold-change value given is 2.0). **d** *Scatter-plot* showing lncRNA expression variation between the control group and LEE011-treated HL-60 cells

The Arraystar Human LncRNA 8 × 60 k v3.01 microarray was used to identify mRNA and lncRNA expression profiles in 1 µM LEE011-treated HL-60 cells compared with a non-treated control group. Microarray analysis and original data have been submitted to the GEO database with accession number GSE81060. In the lncRNA and mRNA expression profiling data we identified 2083 differentially expressed mRNAs in LEE011-treated HL-60 cells compared with the controls. Compared with the control group, 116 mRNAs were upregulated and 155 mRNAs were downregulated at the level of >fivefold change in LEE011 treated HL-60 cells. Clustering analysis of these mRNA expression patterns is presented in Fig. 6a, c and Additional files 3, 4. In lncRNA analysis, 3224 lncRNAs were differentially expressed in LEE011-treated HL-60 cells from a total of 33,327 lncRNAs. Hierarchical clustering analysis of the differently expressed lncRNAs (fold change ≥5) is presented in Fig. 6b, d and Additional files 5, 6.

In our system hundreds of brain-derived neurotrophic factor (BDNF) related lncRNAs were upregulated, including BDNF-AS (NR_033313, NR_002832, ENST00000530313, ENST00000532965) and BDNF-AS1 (uc009yis.3). BDNF plays an important role in the aging process [27]. BDNF helps to protect neurons from damage caused by infection or injury. A study performed in rats showed that TrkB (a BDNF receptor) is markedly decreased during the aging process [28]. DLGAP1 related lncRNAs such as uc002kmi.3, ENST00000573177, uc010wzb.2, uc002kmj.1, NR_024101, ENST00000575606, ENST00000573355 and ENST00000576606 were also upregulated in our system. DLGAP1 plays a fundamental role in centrosome positioning and cell polarity. Centrosome positioning is crucial for cellular senescence [28].

### Gene ontology and KEGG pathway analysis of mRNA expression profiles in LEE011-treated HL-60 cells

**Fig. 7:**
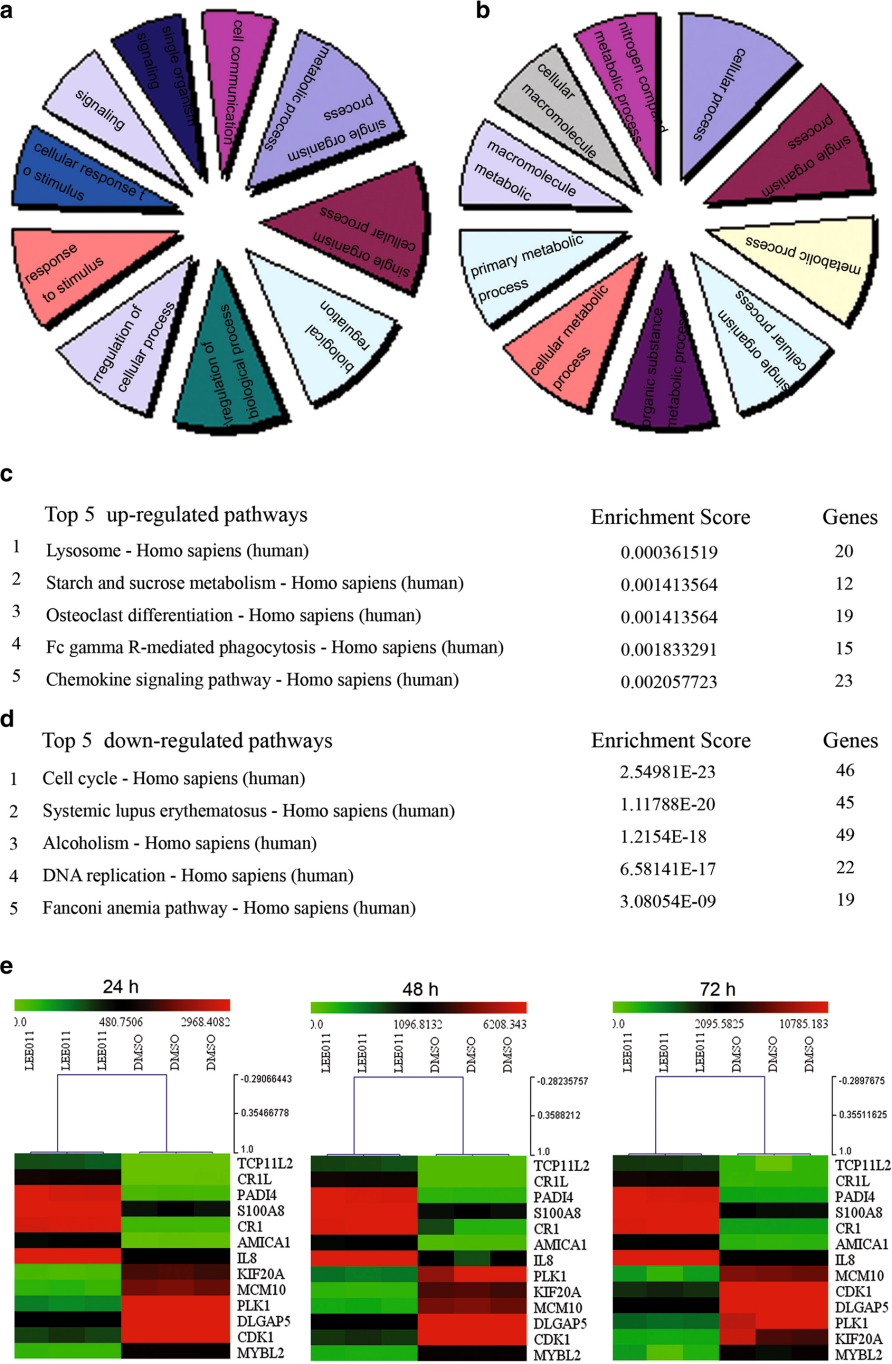
Gene ontology and KEGG pathway analysis of mRNA expression profiles in LEE011-treated HL-60 cells. **a** The most enriched GO terms for upregulated transcripts. **b** The most enriched GO terms for downregulated transcripts. **c** The top five enriched pathways for upregulated transcripts from KEGG pathway analysis. **d** The top five enriched pathways for downregulated transcripts from KEGG pathway analysis. The most enriched pathway was cell cycle, with a P value of 2.54981E^−23^. The cell cycle pathway included BUB1, BUB1B, BUB3, CCNA1, CCNA2, CCNB1, CCNB2, CCNE2 and CDC25A, amongst others. **e** Cluster analysis of several genes whose expression was detected by real-time PCR in HL-60 cells treated with 1uM LEE011 for 24, 48 and 72 h. Gene expression levels for each sample were normalized to the expression level of GAPDH within a given sample (−∆Ct). The relative expression of each gene was calculated using the equation: 10^6^ × log_2_ (−∆Ct). Gene expression differences between the DMSO-treated and the LEE011-treated samples were analyzed using Multi Experiment View (MEV) cluster software

Gene ontology pathway enrichment analysis was performed for the differentially expressed genes identified through microarray analysis. Fisher’s exact test was used to determine whether the differential expression was greater than that expected by chance. For the upregulated transcripts (Fig. 7a), the most enriched gene ontologies (GOs) included immune response (P = 1.69936E^−13^), immune system process (P = 1.71894E^−13^), defense response (P = 3.32706E^−13^) and response to stimulus (P = 2.09266E^−12^). For the downregulated transcripts (Fig. 7b), the most enriched GOs included mitotic cell cycle (P = 1.03536E^−96^), cell cycle (P = 1.62994E^−88^), cell cycle process (P = 7.37652E^−84^) and mitotic cell cycle process (P = 1.36566E^−81^). We further investigated the pathways in which these differentially expressed genes are involved through KEGG database analysis. The five most enriched pathways from KEGG analysis are shown in Fig. 7c, d. Notably, the downregulated pathways included cell cycle and DNA replication pathways.

### LEE011 induced cellular senescence in leukemia cells partially through downregulation of the transcriptional expression of MYBL2

**Fig. 8:**
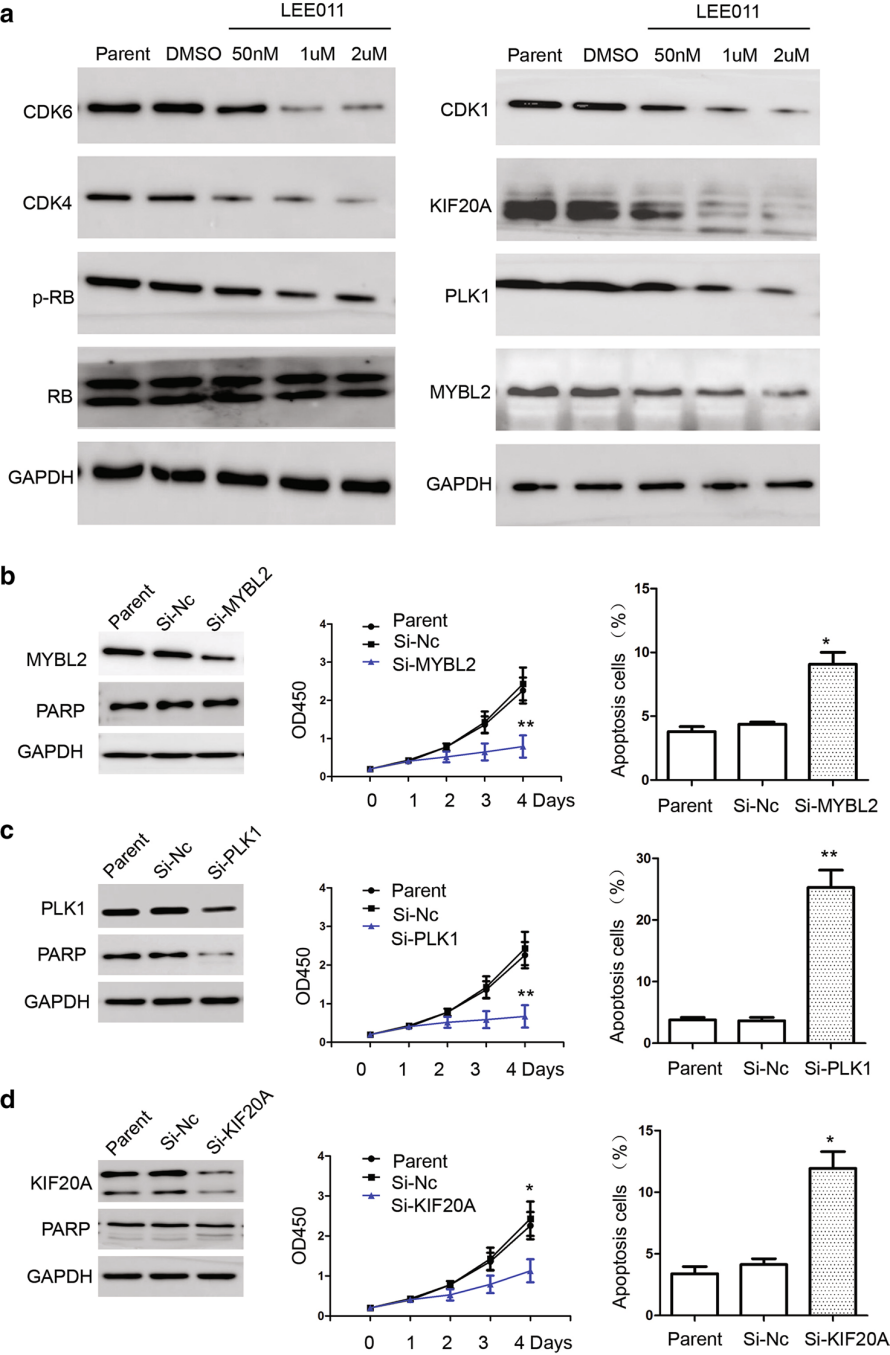
Molecular mechanism analysis of cellular senescence induced by LEE011 in leukemia cells. **a** Dysregulated genes, KIF20A and MYBL2, identified from gene arrays were confirmed by western blot analysis. **b** RNA interference of MYBL2 significantly down regulates the expression of MYBL2 in HL-60 cells. Proliferation analysis of Si-MYBL2 at 4 days showed an OD450 value of 0.79 ± 0.29 vs. Si-Nc 2.43 ± 0.43, P < 0.01. Apoptosis analysis showed Si-MYBL2 was 9.06 ± 1.62% vs. Si-Nc 4.36 ± 0.31%, P = 0.034. **c** RNA interference significantly downregulates the expression of PLK1 in HL-60 cells. Proliferation analysis of Si-MYBL2 at 4 days showed an OD450 value of 0.67 ± 0.29 vs. Si-Nc 2.43 ± 0.43, P < 0.01. Apoptosis analysis showed Si-MYBL2 was 25.26 ± 4.91% vs. Si-Nc 3.67 ± 0.89%, P < 0.01. **d** RNA interference significantly downregulates the expression of KIF20A in HL-60 cells. Proliferation analysis of Si-MYBL2 at 4 days showed an OD450 value of 1.13 ± 0.39 vs. Si-Nc 2.43 ± 0.43, P < 0.05. Apoptosis analysis showed Si-MYBL2 was 11.9 ± 2.35% vs. Si-Nc 4.13 ± 0.81%, P = 0.02. *P < 0.05; **P < 0.01

To identify the cellular senescence molecules implicated in the mechanism of LEE011, the expressions of certain dysregulated genes identified in the gene array were confirmed using real-time PCR and western blot analyses. Cluster analysis of the real-time PCR results showed dysregulated genes in HL-60 cells treated with 1 µM LEE011 for 24–72 h (Fig. 7e). Western blot analysis showed the downregulation of KIF20A, PLK1 and MYBL2 (Fig. 8a). These results are consistent with the real-time PCR analysis. Our western blot analysis also showed that LEE011 treatment could decrease the phosphorylation of RB and expression of CDK4/6 (Fig. 8a).

The molecular function of MYBL2, PLK1 and KIF20A was also analyzed in HL-60 cells. RNA interference of MYBL2 significantly downregulated the expression of MYBL2. Cell proliferation was also inhibited when the expression of MYBL2 was downregulated by RNA interference (Fig. 8b). Figure 8c, d show that downregulation of PLK1 and KIF20A resulted in inhibition of proliferation and induction of apoptosis in HL-60 cells.

**Fig. 9:**
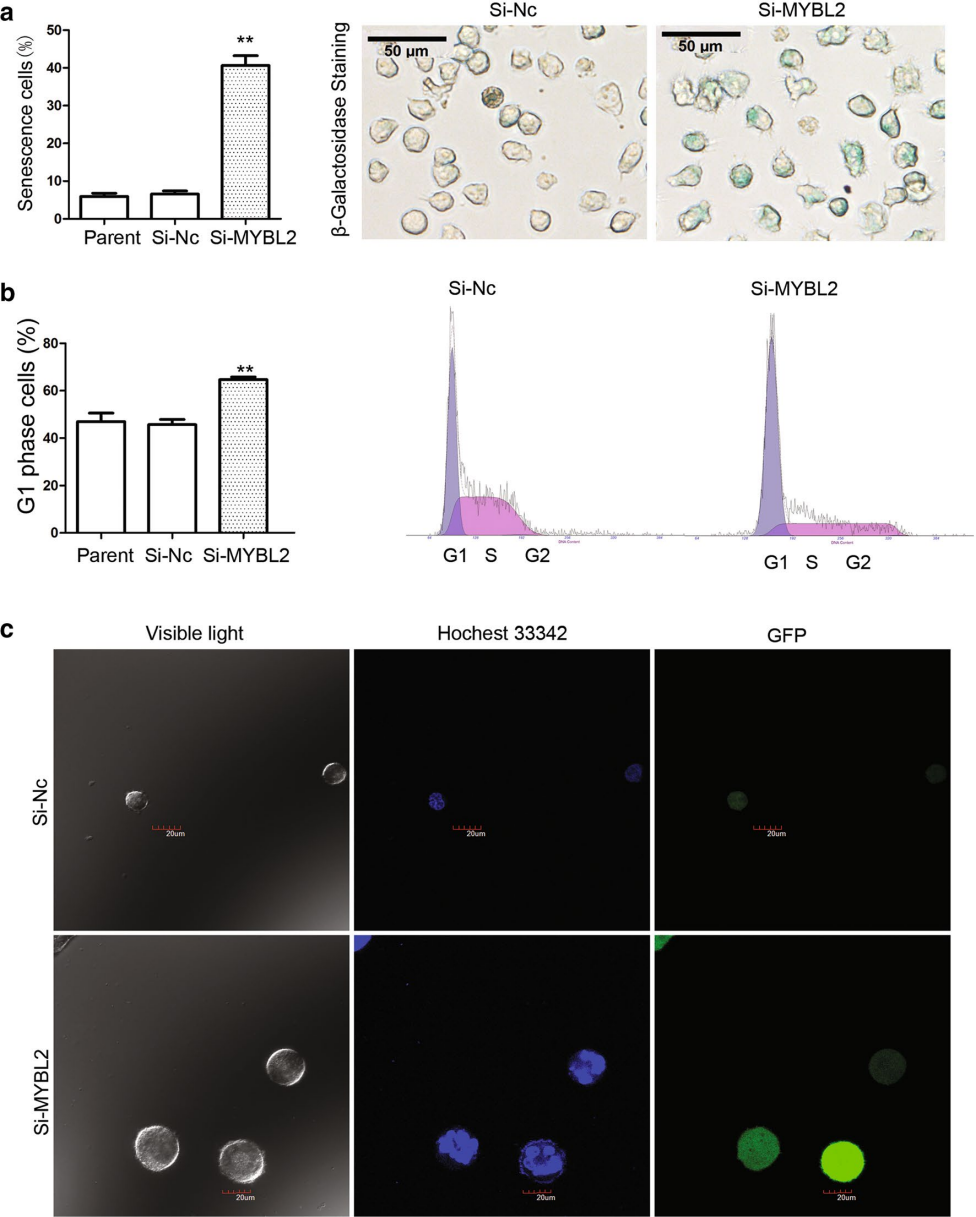
LEE011 induced cellular senescence in leukemia cells partially through downregulation of the transcriptional expression of MYBL2. **a** Cell senescence β-galactosidase staining analysis showed that in the Si-MYBL2 group, positively stained cells increased compared with the Si-Nc control group: Si-MYBL2 group 40.63 ± 4.48% vs. Si-Nc group 6.57 ± 1.42%, P = 0.003. **b** Cell cycle analysis showed that G1 phase cells in the Si-MYBL2 group increased significantly: Si-MYBL2 group 64.67 ± 1.98% vs. Si-Nc group 45.73 ± 3.72%, P = 0.004. **c** DNA staining with Hochest 33,342 showed that nucleus became larger and irregular in the Si-MYBL2 group cells. **P < 0.01

Cell senescence β-galactosidase staining analysis showed that in the Si-MYBL2 group, the number of positive cells was increased compared with the Si-Nc control group (Fig. 9a). Cell cycle analysis showed that G_1_ phase cells in the Si-MYBL2 group increased significantly (Fig. 9b). DNA staining with Hochest 33,342 showed that nucleus became larger and irregular in the Si-MYBL2 group cells (Fig. 9c). These results imply that LEE011 induced senescence in AML cells, partially through down regulation of the transcriptional expression of MYBL2. MYBL2 may be a new target of LEE011, but molecular function analysis of other target genes of LEE011 is still required.

## Discussion

Currently, three selective CDK4/6 inhibitors, palbociclib (PD-0332991), ribociclib (LEE011) and abemaciclib (LY2835219), have been clinically approved or are in late-stage clinical trials [29]. LEE011 (ribociclib) is an orally-applied, effective small molecule that inhibits CDK4/6 at nanomolar concentrations. Antitumor activity of LEE011 has been demonstrated in several cancer models. Sixteen active clinical trials are currently underway with LEE011 as a single agent or in use in combination with other drugs [30, 31]. Most trials with LEE011 are for solid tumors including melanoma, breast cancer and neuroblastoma, and there have been no clinical trials of LEE011 in leukemia or other cancers of the hemopoietic system. In this study, we showed for the first time that LEE011 treatment results in inhibition of cell proliferation and induction of G_1_ arrest and senescence in leukemia cells. The lncRNA microarray was used to determine mRNA and lncRNA expression profiles in LEE011-treated HL-60 cells and demonstrated that LEE011 induced cellular senescence partially through downregulation of the expression of MYBL2.

MYBL2 is emerging as an important gene in cellular senescence. When cells are senescing, MYBL2 has been shown to consistently be the most downregulated gene [32]. As reported previously, ectopic expression of MYBL2 in HMF3A cells can bypass cell senescence [33]. In rodent cells, premature senescence caused by the Ras oncogene can be rescued by MYBL2 expression. Moreover, downregulation of MYBL2 with siRNA silencing leads to increased senescence in primary human foreskin fibroblasts and HeLa cervical cancer cells [33]. These results strongly imply an important role for MYBL2 in senescence. However, it remains to be determined what regulates the expression of MYBL2 and whether MYBL2 could be a novel anti-tumor target [34]. In this study, MYBL2 was downregulated in HL-60 cells treated with LEE011 and cell senescence β-galactosidase staining analysis showed that in Si-MYBL2 cells, positive staining was increased when compared with the Si-Nc control group. Cell cycle analysis showed that G_1_ phase cells increased significantly and nucleus became larger and irregular in the Si-MYBL2 group cells. These results imply that LEE011 induces senescence in AML cells partially through downregulation of the transcriptional expression of MYBL2. Therefore, our study may provide new clues into the mechanism of senescence induced by LEE011 in AML cells.

## Conclusions

In this study, we have shown that LEE011 treatment resulted in inhibition of cell proliferation and induction of G_1_ arrest and cellular senescence in leukemia cells. The lncRNA microarray was used to identify mRNA and lncRNA expression profiles in LEE011 treated HL-60 cells and we demonstrated that LEE011 induces cellular senescence partially through downregulation of the expression of MYBL2. These results may provide new insights into the molecular mechanism of the anticancer effects of LEE011 and its potential as a candidate drug for leukemia; however, further research will be required to determine the underlying details.

## Additional files

**Additional file 1.** Cell cycle analysis of acute leukemia cells treated with LEE011. Cell cycle analysis showed the cells treated with LEE011 2 and 5 μM for 48 hours, results showed in these acute leukemia cells, LEE011 induce the cell cycle G_1_ arrest very significantly. G_1_ phase cells in K562 treated with 5 μM group was 81.23±3.84% vs. DMSO group 32.46±2.21%, P<0.01. G_1_ phase cells in CCRF treated with 5 μM group was 89.83±2.67% vs. DMSO group 45.80±3.24%, P<0.01. G_1_ phase cells in NB4 treated with 5 μM group was 94.79±4.93% vs. DMSO group 45.59±3.12%, P<0.01. G_1_ phase cells in HL-60 treated with 5 μM group was 95.50±2.97% vs. DMSO group 47.40±3.00%, P<0.01. ** *P* < 0.01.

**Additional file 2.** Cell cycle analysis of acute leukemia cells treated with LEE011. Cell cycle analysis showed the cells treated with LEE011 2 and 5 μM for 48 hours, results showed in these acute leukemia cells, LEE011 induce the cell cycle G_1_ arrest very significantly.G_1_ phase cells in MV4-11 treated with 5 μM group was 83.82±2.81% vs. DMSO group 44.66±1.90%, P<0.01. G_1_ phase cells in SHI-1 treated with 5 μM group was 87.39±2.80% vs. DMSO group 45.25±4.61%, P<0.01. G_1_ phase cells in THP-1 treated with 5 μM group was 50.51±8.17% vs. DMSO group 45.64±6.46 %, P=0.466. G_1_ phase cells in U937 treated with 5 μM group was 89.99±3.54% vs. DMSO group 54.26±2.92 %, P<0.01. ** *P* < 0.01.

**Additional file 3.** Differentially Expressed mRNAs up regulated in HL-60 cells treated with LEE011.

**Additional file 4.** Differentially Expressed mRNAs down regulated in HL-60 cells treated with LEE011.

**Additional file 5.** Differentially Expressed LncRNAs up regulated in HL-60 cells treated with LEE011.

**Additional file 6.** Differentially expressed LncRNAs down regulated in HL-60 cells treated with LEE011.

**Additional file 7.** Summary of KEGG analysis the differentially expressed mRNAs in HL-60 cells treated with LEE011.

## Abbreviations

AML: acute myeloid leukemia
LncRNA: long non-coding RNA
GO: gene ontology
KEGG: Kyoto encyclopedia of genes and genomes
GEO: gene expression omnibus
PBMCs: peripheral blood mononuclear cells
