# Supplementary figures and images for: Molecular mechanism of G1 arrest and cellular senescence induced by LEE011, a novel CDK4/CDK6 inhibitor, in leukemia cells
Source: Cancer Cell Int. 2017 Mar 6;17:35. doi: 10.1186/s12935-017-0405-y (PMC5340031; doi:10.1186/s12935-017-0405-y)

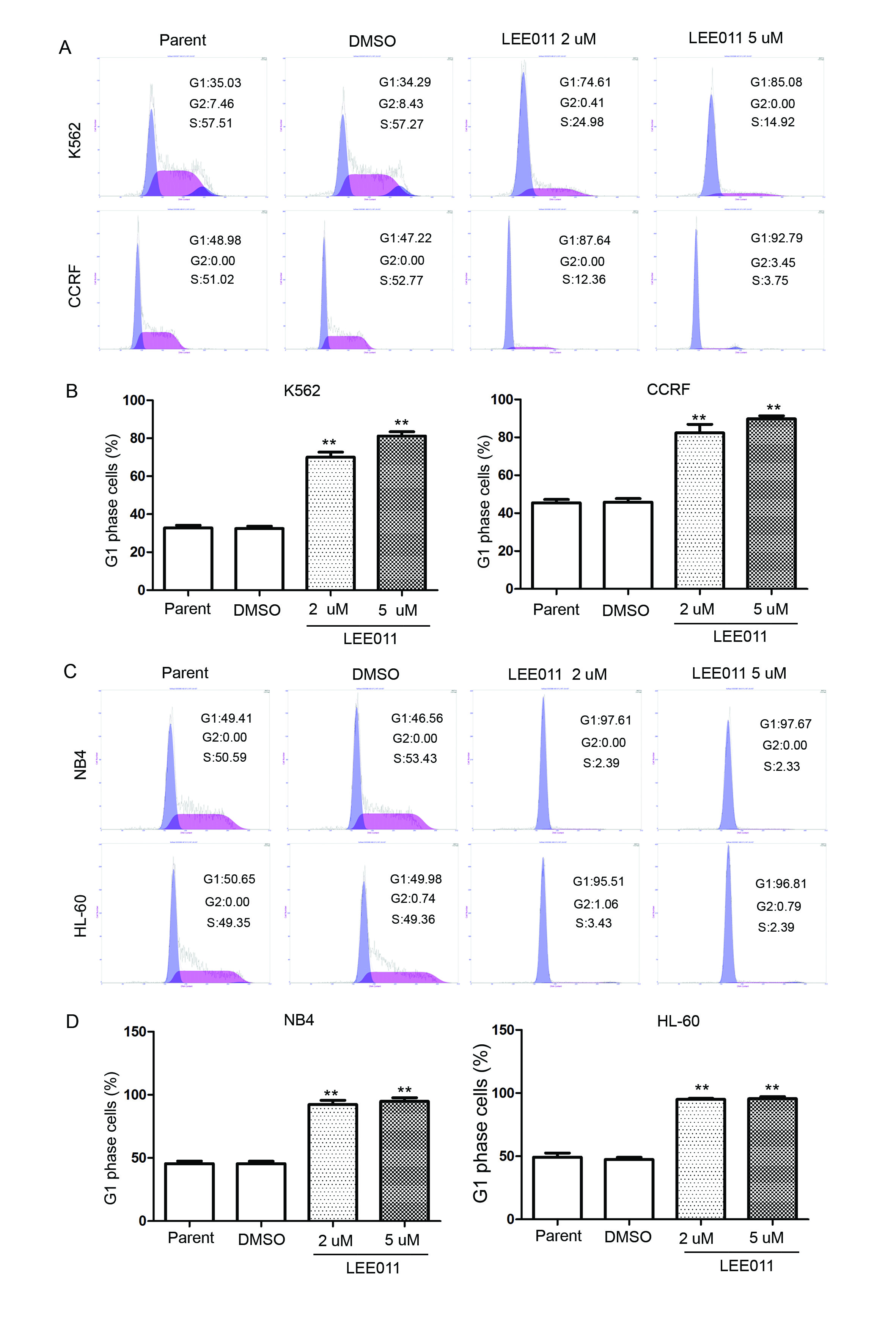

Supplement: Supplementary file 1 — Additional file 1. Cell cycle analysis of acute leukemia cells treated with LEE011. Cell cycle analysis showed the cells treated with LEE011 2 and 5 μM for 48 hours, results showed in these acute leukemia cells, LEE011 induce the cell cycle G1 arrest very significantly. G1 phase cells in K562 treated with 5 μM group was 81.23±3.84% vs. DMSO group 32.46±2.21%, P<0.01. G1 phase cells in CCRF treated with 5 μM group was 89.83±2.67% vs. DMSO group 45.80±3.24%, P<0.01. G1 phase cells in NB4 treated with 5 μM group was 94.79±4.93% vs. DMSO group 45.59±3.12%, P<0.01. G1 phase cells in HL-60 treated with 5 μM group was 95.50±2.97% vs. DMSO group 47.40±3.00%, P<0.01. ** P < 0.01. [file 12935_2017_405_MOESM1_ESM.jpg]

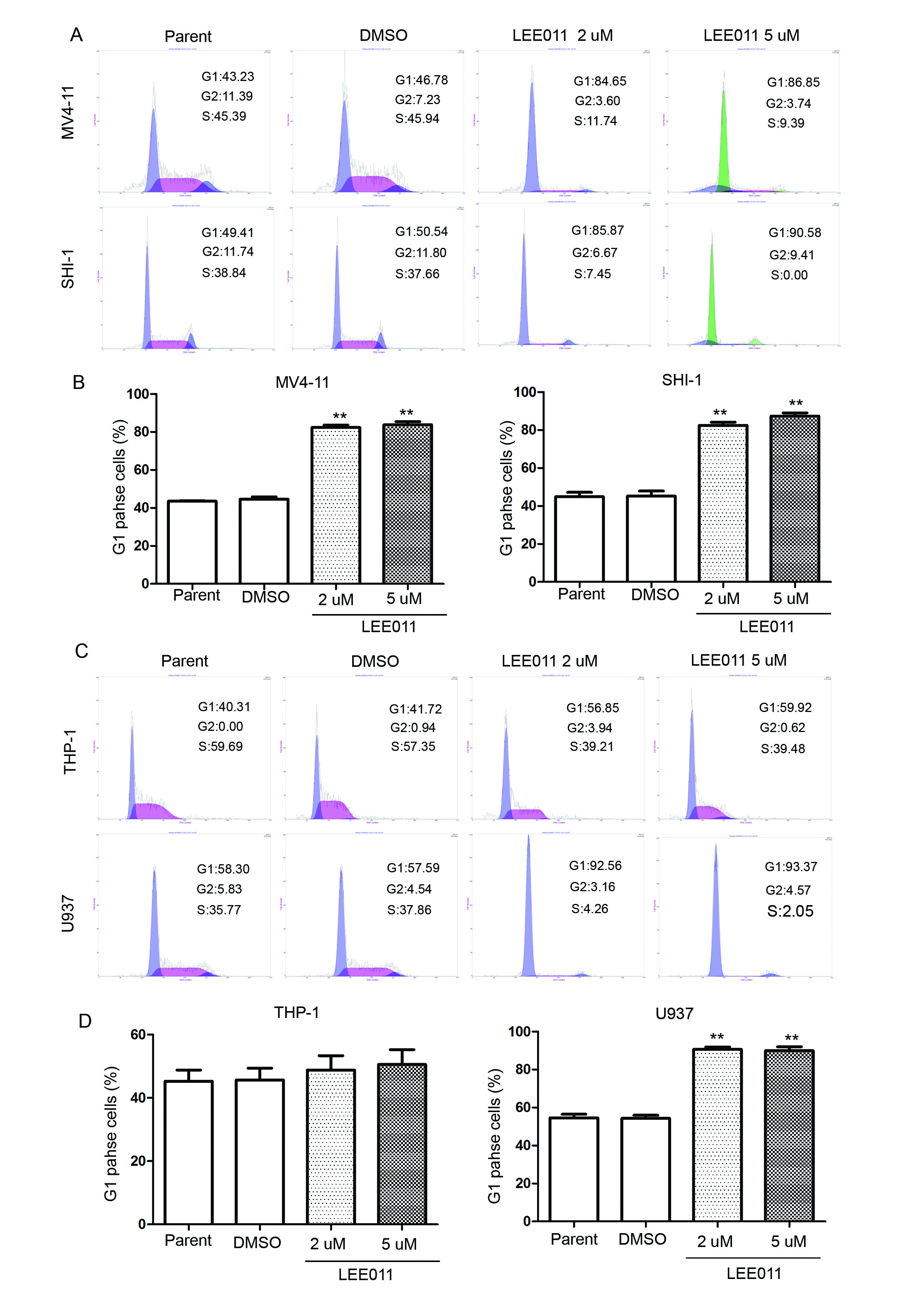

Supplement: Supplementary file 2 — Additional file 2. Cell cycle analysis of acute leukemia cells treated with LEE011. Cell cycle analysis showed the cells treated with LEE011 2 and 5 μM for 48 hours, results showed in these acute leukemia cells, LEE011 induce the cell cycle G1 arrest very significantly.G1 phase cells in MV4-11 treated with 5 μM group was 83.82±2.81% vs. DMSO group 44.66±1.90%, P<0.01. G1 phase cells in SHI-1 treated with 5 μM group was 87.39±2.80% vs. DMSO group 45.25±4.61%, P<0.01. G1 phase cells in THP-1 treated with 5 μM group was 50.51±8.17% vs. DMSO group 45.64±6.46 %, P=0.466. G1 phase cells in U937 treated with 5 μM group was 89.99±3.54% vs. DMSO group 54.26±2.92 %, P<0.01. ** P < 0.01. [file 12935_2017_405_MOESM2_ESM.jpg]
